# The SRC Inhibitor Dasatinib Induces Stem Cell-Like Properties in Head and Neck Cancer Cells that are Effectively Counteracted by the Mithralog EC-8042

**DOI:** 10.3390/jcm8081157

**Published:** 2019-08-02

**Authors:** Francisco Hermida-Prado, M. Ángeles Villaronga, Rocío Granda-Díaz, Nagore del-Río-Ibisate, Laura Santos, Maria Ana Hermosilla, Patricia Oro, Eva Allonca, Jackeline Agorreta, Irati Garmendia, Juan Tornín, Jhudit Perez-Escuredo, Rocío Fuente, Luis M. Montuenga, Francisco Morís, Juan P. Rodrigo, René Rodríguez, Juana M. García-Pedrero

**Affiliations:** 1 Department of Otolaryngology, Hospital Universitario Central de Asturias and Instituto de Investigación Sanitaria del Principado de Asturias; Instituto Universitario de Oncología del Principado de Asturias, University of Oviedo, 33011 Oviedo, Spain; 2 Ciber de Cáncer, CIBERONC, 28029 Madrid, Spain; 3 EntreChem SL, Vivero Ciencias de la Salud, 33011 Oviedo, Spain; 4 Program in Solid Tumors, Center for Applied Medical Research (CIMA), Department of Pathology, Anatomy and Physiology, University of Navarra, and Navarra’s Health Research Institute (IDISNA), 31008 Pamplona, Spain; 5 Division of Pediatrics, Department of Medicine, Faculty of Medicine, University of Oviedo, 33006 Oviedo, Spain

**Keywords:** head and neck squamous cell carcinoma, SRC, dasatinib, saracatinib, cancer stem cells, EC-8042

## Abstract

The frequent dysregulation of SRC family kinases (SFK) in multiple cancers prompted various inhibitors to be actively tested in preclinical and clinical trials. Disappointingly, dasatinib and saracatinib failed to demonstrate monotherapeutic efficacy in patients with head and neck squamous cell carcinomas (HNSCC). Deeper functional and mechanistic knowledge of the actions of these drugs is therefore needed to improve clinical outcome and to develop more efficient combinational strategies. Even though the SFK inhibitors dasatinib and saracatinib robustly blocked cell migration and invasion in HNSCC cell lines, this study unveils undesirable stem cell-promoting functions that could explain the lack of clinical efficacy in HNSCC patients. These deleterious effects were targeted by the mithramycin analog EC-8042 that efficiently eliminated cancer stem cells (CSC)-enriched tumorsphere cultures as well as tumor bulk cells and demonstrated potent antitumor activity in vivo. Furthermore, combination treatment of dasatinib with EC-8042 provided favorable complementary anti-proliferative, anti-invasive, and anti-CSC functions without any noticeable adverse interactions of both agents. These findings strongly support combinational strategies with EC-8042 for clinical testing in HNSCC patients. These data may have implications on ongoing dasatinib-based trials.

## 1. Introduction

Increased SRC expression and/or activity has been widely detected in a variety of human cancers, including head and neck squamous cell carcinomas (HNSCC) [1,2,3]. It has been demonstrated that aberrant SRC activity plays a central role in all stages of tumorigenesis from malignant transformation to tumor progression and ultimately development of metastatic disease [4,5].

SRC is a pleiotropic non-receptor tyrosine kinase that interacts with multiple receptor tyrosine kinases and modulates multiple oncogenic signaling pathways [6,7], thereby regulating a variety of cellular processes central to the malignant phenotype, including proliferation, survival, cell-cell adhesion, motility, invasion, and angiogenesis [8,9]. SRC induces disruption of focal adhesions by activation of focal adhesion kinase (FAK) [10]. FAK is associated with SRC in focal adhesions, which results in the phosphorylation of FAK followed by downstream activation of the Ras-mediated pathways [11]. In addition, SRC regulates the reorganization of the actin cytoskeleton through phosphorylation of p190 [12] and cortactin (CTTN), which results in increased motility [13].

The frequent dysregulation of SRC family kinases (SFK) in a variety of human cancers has led to a rapid development of multiple agents aimed at targeting SFK in cancer treatment [9]. Various inhibitors, in particular dasatinib (BMS-354825) and saracatinib (AZD0530), have been the subject of intense research in recent years [14,15,16,17,18,19,20,21]. Even though both drugs have proved powerful as antitumor agents in preclinical settings, their clinical efficacy in cancer patients has been limited if not disappointing [14,15,16,20,21]. In preclinical models of HNSCC, dasatinib and saracatinib showed potent effects on proliferation, cell migration, and invasion [22,23]; however, both inhibitors failed to demonstrate any significant activity as single agents in patients with recurrent and/or metastatic HNSCC [24,25].

In light of these data, it becomes highly desirable to establish relevant response biomarkers to improve patient stratification, treatment efficacy, and ultimately clinical outcome. In addition, a deeper functional and mechanistic knowledge of the actions of these drugs on tumor cells will contribute to deciphering not only anti-tumor functions but also unwanted pro-tumor actions, thus enabling the development of combination strategies that may prevent potential deleterious activities and provide anti-tumor complementary/synergistic strategies.

This study demonstrates that while dasatinib and saracatinib robustly blocked cell migration and invasion in HNSCC cell lines, both were found to strikingly enhance cancer stem cells (CSC) properties. These deleterious effects were effectively targeted by the low-toxicity mithramycin analog (mithralog) EC-8042 [26], which has previously demonstrated anti-stemness activity in other cancer cells [27]. Accordingly, EC-8042 efficiently inhibited the growth of both bulk tumor adherent cultures and CSC-enriched tumorsphere cultures in HNSCC-derived cells and demonstrated a robust antitumor activity in vivo. Moreover, combination treatment between dasatinib and EC-8042 benefits from complementary anti-tumor properties provided by each compound without any noticeable adverse interactions between them.

## 2. Materials and Methods

### 2.1. Drugs

EC-8042 (EntreChem, Oviedo, Spain), Dasatinib and Saracatinib (both from Selleck, Suffolk, UK) were prepared as 10 mM solutions in sterile DMSO or water, maintained at −20 °C and brought to the final concentration just before use.

### 2.2. Cell Culture

FaDu cells (male, hypopharyngeal squamous cell carcinoma) were purchased from the ATCC, and the HNSCC cell line UT-SCC38 derived from a laryngeal squamous carcinoma (T2N0M0) was kindly provided by R. Grenman (Department of Otolaryngology, University Central Hospital, Turku, Finland) [28]. Cells were grown in DMEM supplemented with 10% fetal bovine serum (FBS), 100 U/mL penicillin, 200 mg/mL streptomycin, 2 mmol/L L-glutamine, 20 mmol/L HEPES (pH 7.3), and 100 mmol/L non-essential amino acids. All the cells derived from HPV-negative primary HNSCC. All cell lines were periodically tested for mycoplasma contamination by PCR using the Biotools Detection kit (Madrid, Spain) specifically amplifying a conserved region of the mycoplasma 16S RNA gene. Cell line authentication was carried out by DNA (STR) profiling at the SCT Core Facilities (University of Oviedo, Spain).

### 2.3. Western Blotting Analysis

Cells were lysed in Laemmli sample buffer and sonicated before centrifugation. Protein lysates were separated by SDS–polyacrylamide gel electrophoresis (SDS–PAGE) and transferred to nitrocellulose membranes (Amersham Protran, GE Healthcare, Pittsburg, PA, USA). Subsequently, membranes were blocked for 1 h with Odyssey blocking buffer (LI-COR Biosciences, Lincoln, NE, USA) and incubated overnight with the indicated primary antibodies (Supplementary Methods) at 1:1000 dilution. The IRDye Infrared Fluorescent secondary antibodies anti-Rabbit and anti-Mouse IRDye 800CW and IRDye 680RD (LI-COR Biosciences) were used for detection. Membranes were scanned with the Odyssey Fc Dual-Mode Imaging System (LI-COR Biosciences) using the red (700 nm) and green (800 nm) channels, and signal analysis was performed using Image Studio Lite software (LI-COR, Nebraska). Results were normalized to GAPDH as loading control.

### 2.4. Cell Viability Assays

HNSCC cells were seeded into 96-well culture plates at a density of 2000 cells per well and incubated overnight. Drugs were serially diluted in medium over a range of concentrations and added to the cells. After 72 h treatment, cell viability was measured in quadruplicates using a MTS assay (CellTiter 96 Aqueous One Solution Cell Proliferation Assay from Promega, Madison, WI, USA) reading absorbance at 490 nm using a Synergy HT plate reader (BioTek, Winooski, VT, USA). The existence of synergy in drug combinations was determined by calculating the combination index (CI) as described in Supplementary Information.

### 2.5. Scratch-Induced Directional Migration Assay

Cells were plated in 24-well dishes with ibidi^®^ culture inserts (ibidi LLC, Verona, WI, USA) at 80–90% confluence, and cell migration monitored as we previously described [29].

### 2.6. Three-Dimensional Spheroid Invasion Assays

Invasion assays using 3D spheroids were performed as previously described [30]. Cells were suspended in DMEM plus 5% Methyl cellulose (Sigma, St Louis, MO, USA) to form cell spheroids by serial pipetting into a non-adhesive Petri dish (2000 cells/spheroid), followed by overnight incubation in an inverted position. The next day, each cell spheroid was individually transferred to a 96-well plate, embedded into bovine collagen matrix (Advanced Biomatrix PureCol), and filled with 100 μL of complete media containing or not containing drugs. Cell invasion was monitored using a Zeiss Cell Observer Live Imaging microscope (Zeiss, Thornwood, NY, USA) and images acquired every 15 min for 24 h using a Zeiss AxioCam MRc camera. The invasive area was calculated as the difference between the final area (t = 24 h) and the initial area (t = 0 h) using image J analysis program, and data were normalized to control (vehicle-treated) cells. Three independent experiments were performed using quadruplicates for each condition.

### 2.7. Tumorsphere Formation Assay

HNSCC-derived cells lines were plated at a density of 500 cells/mL in 6-well tissue culture plates treated with a sterile solution of polyHEMA (10 g/L in 95% ethanol) (Sigma) to prevent cell attachment. Cells were grown in DMEM-F12 (GE Healthcare) supplemented with 1% Glutamax and 2% B27 Supplement from Life Technologies (Rockford, IL, USA), 10 ng/mL human bFGF and 20 ng/mL human EGF (PeproTech, London, UK) and 100 U/mL penicillin and 200 mg/mL streptomycin (Thermo, Waltham, MA, USA).

After 10–12 days, well-formed spheres were photographed in Leica Microsystems microscope DMIL T coupled with a Leica DC500 High-resolution Digital Camera (Leica Microsystems, Barcelona, Spain). Then, the tumorspheres were centrifuged at 300 rpm for 2 min, washed with PBS and either collected for RNA extraction or disaggregated with Gibco trypsin (0.25%)/EDTA for 15 min to measure cell viability by MTS.

### 2.8. RNA Extraction and Real-Time RT-PCR

Total RNA was extracted from HNSCC cells using Trizol reagent (Invitrogen Life Technologies, Carlsbad, CA, USA), and gene expression was analyzed by real-time RT-PCR as we previously reported [31] using SYBR Green Master Mix protocol (Applied Biosystems, Foster City, CA, USA) in a StepOnePlus Real-Time PCR System (Applied Biosystems, Foster City, CA, USA). Reactions were run in triplicates using the specific primers detailed in Supplementary Table S1, and the ribosomal coding gene RPL19 was used as endogenous control. The relative mRNA expression was calculated using the 2^−ΔΔ*C*T^ method, and the data were expressed as the fold-change normalized to RPL19 mRNA levels and relative to control (vehicle-treated) cells.

### 2.9. In Vivo Treatments of FaDu Xenografts

All experimental protocols were performed in accordance with the institutional guidelines of the University of Oviedo and approved by the Animal Research Ethical Committee of the University of Oviedo prior to the study. Female athymic NMRI-nude mice of 6–7 weeks old (Janvier Labs, St Berthevin, France) were subcutaneously inoculated (s.c.) with 1.5 × 10^6^ FaDu cells mixed 1:1 with BD Matrigel Matrix High Concentration (BD Biosciences, Erembodegem, Belgium) previously diluted 1:1 in culture medium. Once tumors reached ~200 mm^3^, mice were randomized into four treatment groups (*n* = 10 per group): (i) vehicle (saline solution intravenously (i.v.) as vehicle for EC-8042 and tartaric acid solution orally for dasatinib); (ii) dasatinib (10 mg/kg every day (16 doses) orally); (iii) EC-8042 (50 mg/kg every 7 days (4 doses) i.v.); and (iv) dasatinib plus EC-8042 combination.

Survival was represented using Kaplan–Meier analysis and the log-rank test to estimate significant differences among groups (PAST 3.01 software, University of Oslo, Norway). Tumor growth and drug efficacy (expressed as the percentage of tumor growth inhibition, %TGI) were calculated as indicated in Supplementary Information.

### 2.10. Tumorsphere Formation and Immunohistochemical Analyses of Tumors from FaDu Xenografts.

Upon removal, tumor samples were weighted and a portion of some tumors was disaggregated into single cell suspensions using MACS Tissue Dissociation Kit and the GentleMACS Dissociator system (Miltenyi Biotec, Bergisch Gladbach, Germany) as previously described [27], in order to perform tumorsphere formation assays after in vivo treatments. The remaining portion of the tumors were fixed in formol, embedded in paraffin, cut into 4-μm sections, and stained with hematoxylin and eosin (H&E). Immunohistochemical analyses were performed in an automatic workstation (Dako Autostainer Plus) with anti-Ki67 (Clone MIB-1 Dako # JR626, Prediluted), anti-active PARP (Abcam # 32064, at 1:500), anti-ALDH1 (BD Biosciences # 611195, at 1:500), anti-SOX2 (Merck Millipore # AB5603, at 1:1000), and phospho-FAK (Y861) (Invitrogen # 44-626G, at 1:100) using the Dako EnVision Flex + Visualization System (Dako Autostainer). The number of ALDH1-positive cells or SOX2-positive nuclei was counted at 40× in five independent microscopic fields per tissue section, and the mean of five fields was calculated for each treatment. p-FAK (Y861) staining intensity was evaluated, and the mean of five fields was calculated for each treatment. Quantification of staining for Ki67 proliferation index (number of positive cells per mm^2^) and cleaved PARP (number of positive cells per mm^2^) was automatically performed using the ImageJ software (National Institutes of Health, Bethesda, MD, USA) in six random images (×200) per sample.

### 2.11. Statistical Analyses

Statistical analysis was performed using GraphPad Prism version 6.0 (Graphpad Software Inc, La Jolla, CA, USA). Data are presented as the mean ± standard deviation (SD) of at least three independent experiments unless otherwise stated. Statistical significance will be determined either using a Student’s unpaired *t*-test with two-tailed distribution for comparison across two groups or one/two-way ANOVA for comparing multiple samples/variables. In comparisons with control groups, the values of *p* < 0.05 were considered statistically significant (* *p* < 0.05; ** *p* < 0.01; *** *p* < 0.001; **** *p* < 0.0001).

## 3. Results

### 3.1. Dasatinib and Saracatinib Completely Blocked Migration and Invasion in HNSCC-Derived Cell Lines

**Figure 1:**
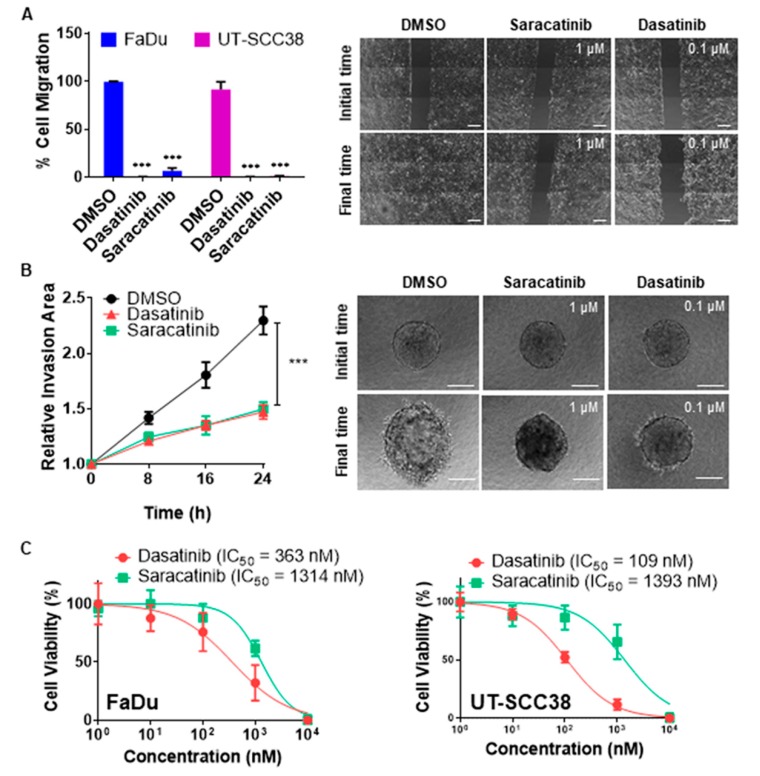
Effect of dasatinib and saracatinib on cell migration, invasion, and growth of head and neck squamous cell carcinomas (HNSCC)-derived cell lines. (**A**) Wound healing assays in FaDu and UT-SCC38 cells treated with either DMSO (vehicle), 0.1 µM dasatinib, or 1 µM saracatinib. The percentage of cell migration (left panel) and representative images showing the initial scratch (t = 0) area and the residual area at the final time (t = 15 h) in FaDu cells (right panel) are displayed. Scale bars = 200 µm. Data are expressed relative to vehicle-treated cells (mean ± SD, Student’s *t*-test, *** *p* < 0.001). (**B**) 3D spheroid invasion assays in FaDu cells treated for 24 h with either DMSO (vehicle), 0.1 µM dasatinib, or 1 µM saracatinib. The quantification of the invasive area at the indicated times (left panel) and representative images of FaDu spheroids at initial (t = 0) and final time (t = 24 h) (right panel) for the different treatments are displayed. Scale bars = 200 μm. Data are expressed relative to DMSO-treated cells (mean ± SD, Student’s *t*-test, *** *p* < 0.001). (**C**) Cell viability of FaDu (left panel) and UT-SCC38 (right panel) cells was measured by MTS assay after 72 h treatment with increasing doses of either dasatinib or saracatinib. IC_50_ values are shown.

We first evaluated the effect of dasatinib and saracatinib in the HNSCC-derived cell lines FaDu and UT-SCC38. As expected, both compounds decreased the phosphorylation levels of SRC at tyrosine 418 (Y418) and FAK at Y861 in FaDu and UT-SCC38 cells (Supplementary Figure S1A,B). Phospho-SRC Y418 levels rapidly decreased after 1 h treatment with saracatinib and dasatinib (Supplementary Figure S1C), and the phosphorylation levels of its downstream target FAK Y861 were efficiently targeted and durably reduced at 24 h. In addition, dasatinib (0.1 µM) and saracatinib (1 µM) completely blocked cell migration and invasion into 3D collagen matrices in both cell lines (Figure 1A,B, and Supplementary Materials: Videos S1 and S2). 24 h treatment with these concentrations of drugs had no significant effect on cell viability in UT-SCC38 and led to a 20% decrease in FaDu cells (Supplementary Figure S1D); however, this effect was very modest compared to the robust effects observed on cell migration (>90%) and invasion (>70%). Altogether, these data indicate that the potent anti-invasive effect observed upon dasatinib or saracatinib treatment cannot be attributed to the ability of these drugs to decrease cell viability. Nonetheless, longer treatments for 72 h with saracatinib and dasatinib led to a dose-dependent reduction of cell viability, with dasatinib having a more pronounced cytotoxic effect (Figure 1C).

### 3.2. Dasatinib and Saracatinib Promoted CSC-Like Phenotypes in HNSCC Cells

CSCs have been recognized to play critical roles driving tumor initiation, progression, recurrence, and treatment resistance. This prompted us to investigate whether dasatinib and saracatinib are able to target CSCs in HNSCC cells.

**Figure 2:**
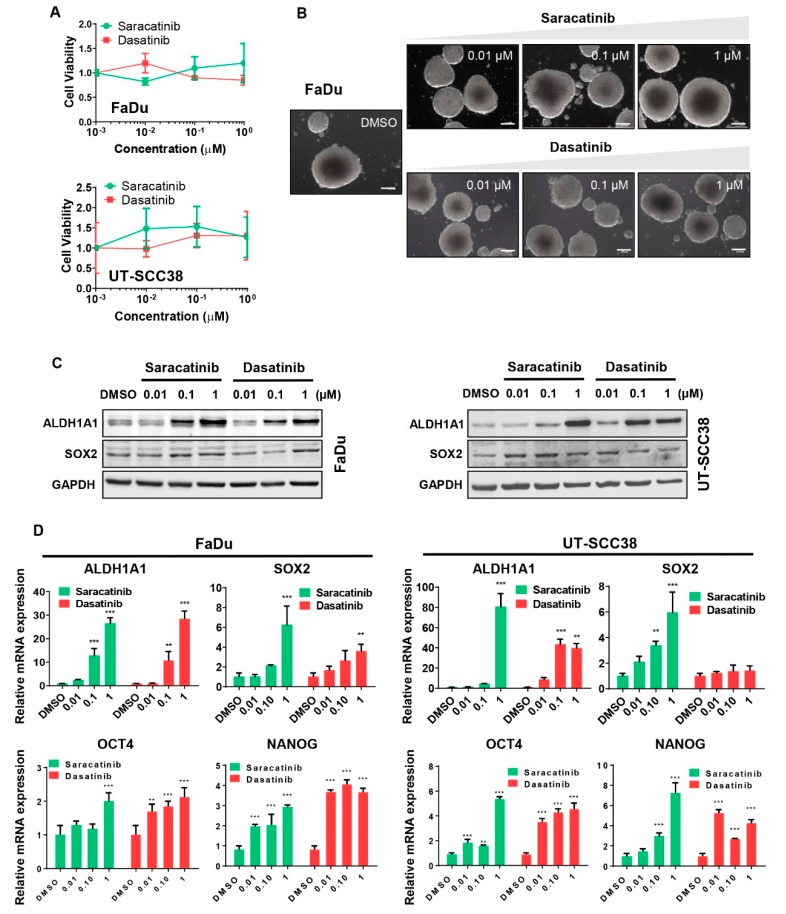
Effect of dasatinib and saracatinib on the cancer stem cells (CSC) properties of HNSCC-derived cell lines. (**A**,**B**) Cell viability (MTS assay) of UT-SCC38 and FaDu tumorspheres treated with increasing concentrations of dasatinib or saracatinib for 72 h. Quantification of cell viability (**A**) and representative images of FaDu tumorspheres upon the indicated treatments (**B**) are shown. (**C**,**D**) Analysis of the protein (Western blotting; C and quantification of IRdye fluorescent signals are plotted in Supplementary Figure S3A) and relative mRNA (RT-qPCR); (**D**) levels of the CSC-markers ALDH1A1, SOX2, OCT4, and NANOG in FaDu and UT-SCC38 cells treated with increasing concentrations of dasatinib and saracatinib for 72 h. Data were normalized to RPL19 levels and represented relative to vehicle-treated cells (mean ± SD, Student’s *t*-test, ** *p* < 0.01, *** *p* < 0.001).

Clonal sphere-forming ability in non-adherent serum-free culture conditions (tumorsphere cultures) is a hallmark of self-renewal and CSC-related phenotype. Under these conditions, the expression of well-known CSC markers such as ALDH1A1 and SOX2 [32] was consistently and markedly enriched in FaDu and UT-SCC38 tumorspheres when compared to unselected adherent cultures (Supplementary Figure S2). Notably, dasatinib and saracatinib failed to eliminate CSC-enriched tumorsphere cultures of FaDu and UT-SCC38 cells (Figure 2A,B). Strikingly, we also found that both drugs significantly increased the expression of ALDH1A1 and SOX2 at both mRNA and protein levels (Figure 2C,D and Supplementary Figure S3A). Of note, specifically in UT-SCC38 cells, SOX2 expression was not affected by dasatinib, and consistent results were obtained at both mRNA and protein level. Nonetheless, NANOG1 and OCT4 levels also consistently increased upon dasatinib and saracatinib treatment in these two HNSCC-derived cell lines (Figure 2D), in good agreement with the previous paper by Koo et al. [33]. Furthermore, since the process of epithelial to mesenchymal transition (EMT) is known to play a critical role as a driver of tumor cell invasion and metastatic spreading in HNSCC [34,35,36], expression changes of EMT markers were also assessed. Our results consistently showed that the expression levels of the epithelial marker E-Cadherin increased upon treatment with dasatinib and saracatinib, while the expression of mesenchymal markers such as Vimentin and Snail decreased, thereby indicating that both compounds inhibit EMT in these two HNSCC cell lines (Supplementary Figure S4). In light of these data, the ability of dasatinib and saracatinib to revert EMT could be an additional mechanism (together with other invasion regulators targeted, e.g., SRC, FAK) to explain the potent inhibitory effect on cell invasiveness exhibited by these drugs.

### 3.3. The Mithramycin Analog EC-8042 Effectively Abrogated the CSC Properties of HNSCC Cells

We next assessed the ability of the mithramycin analog EC-8042 to eradicate CSCs, since it has been described to potently reduce CSCs viability and expression of CSC-related markers in other cancers [27].

**Figure 3:**
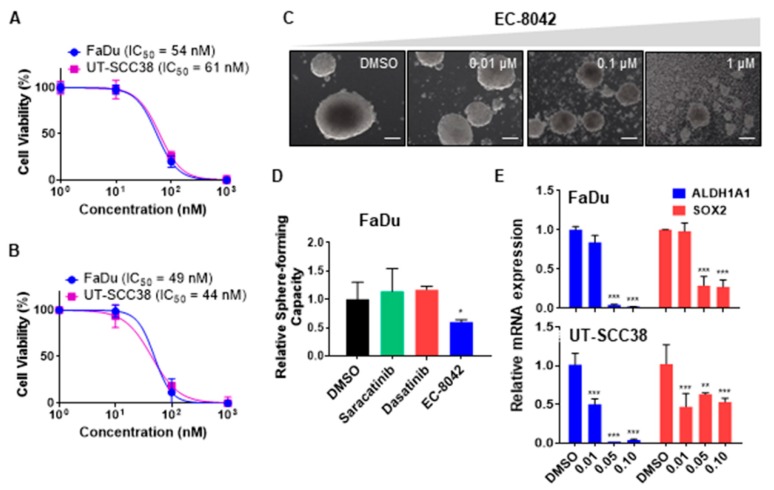
Effect of the mithralog EC-8042 on the viability and CSC properties of HNSCC-derived cell lines. (**A**,**B**) Dose-response curves of the cell viability by MTS in UT-SCC38 and FaDu adherent cells (**A**) or CSC-enriched tumorspheres (**B**) treated with increasing concentrations of EC-8042 for 72 h. IC_50_ values are shown. (**C**) Representative images of FaDu tumorspheres upon treatment for 72 h with different doses of EC-8042. (**D**) Tumorsphere-forming ability of FaDu cells upon treatment for 72 h with either DMSO (vehicle), 0.1 µM dasatinib, 1 µM saracatinib, or 0.1 µM EC-8042. (**E**) RT-qPCR analysis of the CSC-markers ALDH1A1 and SOX2 in adherent cultures of FaDu and UTSCC38 cells treated with increasing concentrations of EC-8042 for 72 h. Data are expressed relative to DMSO-treated cells (mean ± SD, Student’s *t*-test, * *p* < 0.05, ** *p* < 0.01, *** *p* < 0.001).

EC-8042 showed a much more robust cytotoxic effect on adherent cultures than the two SRC inhibitors (Figure 3A). Likewise, the treatment with EC-8042 was highly effective in reducing the number, size and the viability of CSC-enriched tumorsphere cultures (Figure 3B,C). Furthermore, pretreatment for 72 hours with EC-8042 (0.1 µM) but neither dasatinib (0.1 µM) nor saracatinib (1 µM) was also able to significantly prevent tumorsphere formation in FaDu cells (Figure 3D). In addition, the expression of the CSC-related markers ALDH1A1 and SOX2 was robustly and consistently reduced in both FaDu and UT-SCC38 cells after treatment with EC-8042 (Figure 3E).

### 3.4. Combined Effects of Treatment with Dasatinib and EC-8042 in HNSCC Models

**Figure 4:**
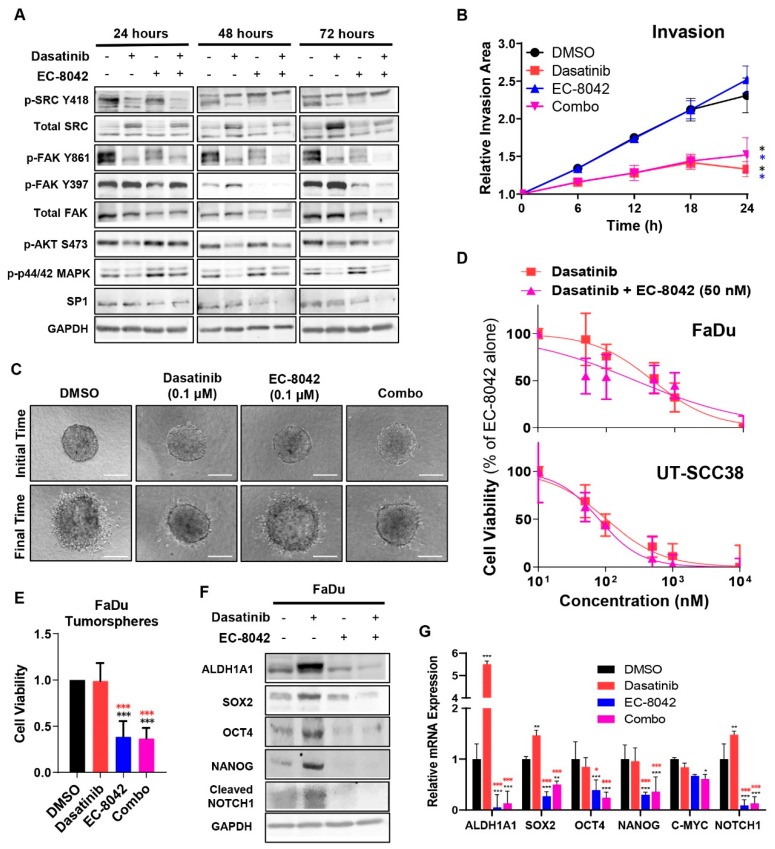
Functional effects of the combined treatment with dasatinib and EC-8042 in HNSCC cells. (**A**) Western blotting analysis of the expression/phosphorylation levels of the indicated proteins in FaDu cells treated for 24 h, 48 h, or 72 h with either 0.1 µM dasatinib or 0.1 µM EC-8042 alone, or in combination. Quantification of IRdye fluorescent signals are plotted in Supplementary Figure S5. (**B**,**C**) 3D spheroid invasion assay in FaDu cells treated for 24 h with 0.1 µM dasatinib, 0.1 µM EC-8042 alone, or combination. The quantification of the invasive area at the indicated times (**B**) and representative images of FaDu spheroids (**C**) at initial (t = 0) and final time (t = 24 h) for the different treatments are shown. Scale bars = 200 μm. Data are expressed relative to vehicle-treated cells (mean ± SD, Repeated Measures (RM)-one-way ANOVA, Tukey’s test, * *p* < 0.05 vs. DMSO and EC-8042-treated cells). (**D**) Dose-response curves of cell viability by MTS in FaDu (upper panel) and UT-SCC38 (lower panel) cells treated with the indicated combinations of drugs for 72 h. In these graphs the dasatinib + EC-8042 series was normalized to the value observed after treatment with EC-8042 alone. The effect of EC-8042 alone is subtracted from the combination values, thus showing the shift in the IC_50_ of dasatinib due to the combination. (**E**) Cell viability of CSC-enriched FaDu tumorspheres treated with 0.1 µM dasatinib, 0.1 µM EC-8042, or combination. Data are expressed relative to vehicle-treated cells (mean ± SD, one-way ANOVA, Tukey’s test, *** *p* < 0.001 vs. DMSO and dasatinib-treated cells). (**F**) Western blotting (left panel) and (**G**) RT-qPCR analysis (right panel) of the indicated oncogenic and CSC-related markers in adherent cultures of FaDu cells treated for 72 h with 0.1 µM dasatinib or 0.1 µM EC-8042 alone, or in combination. mRNA levels were normalized to RPL19 levels and the relative fold-change to vehicle-treated cells ± SD plotted, two-way ANOVA, Dunnett’s test, * *p* < 0.05, ** *p* < 0.01, *** *p* < 0.001 vs. DMSO (black) and dasatinib-treated cells (red). Quantification of the IRdye fluorescent signals are plotted in Supplementary Figure S3B.

Next, we explored the effects of the combination treatment with dasatinib and EC-8042. First, we analyzed the effect of the combination on the activation of key signaling targets for both drugs (Figure 4A and Supplementary Figure S5). As shown previously in other tumor cell models [37], a 24-hour treatment with dasatinib efficiently inhibited SRC phosphorylation at Y418 while upregulating total SRC levels. SRC-dependent phosphorylation and activation of FAK at Y861, but not SRC-independent autophosphorylation at Y397, was completely and durably inhibited by dasatinib without affecting total FAK levels. The phosphorylation/activation of other downstream signaling targets, such as AKT at S473 or p44/42-MAPK, was also inhibited by dasatinib treatment (Figure 4A). EC-8042 treatment resulted in reduced levels of its target SP1 and also led to the down-regulation of both total FAK and p-FAK (Y397) levels. Importantly, the combination of both drugs produced an additive inhibitory effect on the phosphorylation/activation of most of the targets (Figure 4A).

We next studied the ability of the combination to add jointly favorable anti-tumor effects with respect to single treatments. First, we found that the combined treatment maintained the ability of dasatinib to abolish invasion of HNSCC cells into 3D collagen matrices (Figure 4B,C, Supplementary Videos S1–S4). In addition, cell survival curves normalized to the effect of EC-8042 alone showed that the combination of dasatinib with EC-8042 did not produce a significant shift of the dasatinib IC_50_ values (Figure 4D). Furthermore, combination index values calculated according to the Chou and Talalay method [38] for EC-8042 and dasatinib combination were close to 1 (Supplementary Figure S6A), thus suggesting that this combination produce additive, rather than synergistic cytotoxic effects.

Moreover, the deleterious effects caused by dasatinib sustaining CSC-like phenotypes were effectively counteracted by EC-8042 co-treatment, thereby reducing the viability of CSC-enriched tumorspheres (Figure 4E) and the expression of various CSC-related factors such as ALDH1, SOX2, NANOG, OCT4, c-MYC, or NOTCH1 at mRNA and protein level (Figure 4F,G and Supplementary Figure S3B).

In addition, the effects of EC-8042 and combo treatments on the targeting of CSC subpopulations were also monitored by flow cytometry using the SORE6 reporter system, which allows dynamic monitoring of CSC subpopulation based on SOX2/OCT4 expression. This system has previously demonstrated its ability to detect CSC subpopulations in different tumor types [39]. Thus, we generated HNSCC-derived cell lines with stable expression of the SORE6 construct (UT-SCC38-SORE6-GFP and FaDu-SORE6-GFP) or their corresponding controls without the SORE6 response element (UT-SCC38-minCMV-GFP and FaDu-minCMV-GFP), which have been used as gating controls in flow cytometry analyses. FaDu-SORE6-GFP and UT-SCC38-SORE6-GFP displayed 28% and 22% of SORE6-positive cells, respectively. As expected for bona fide CSCs subpopulations, SORE-GFP+ subpopulations sorted from both cell lines by flow cytometry showed increased expression of SOX2 and OCT4 and also increased ability to grow as tumorspheres (Supplementary Figure S7A,B). To analyze the effect of the drugs on CSC subpopulations, cultures of SCC38-SORE6-GFP and FaDu-SORE6-GFP cells were treated with dasatinib, EC-8042 and the combination for 72 h. As shown by flow cytometry analysis, EC-8042 and the combined treatment, but not dasatinib alone, significantly decreased the SORE-GFP+ subpopulation in both cell lines (Supplementary Figure S7C,D), thus confirming the ability of EC-8042 alone or in combination with dasatinib to target CSC subpopulations in HNSCC cells.

**Figure 5:**
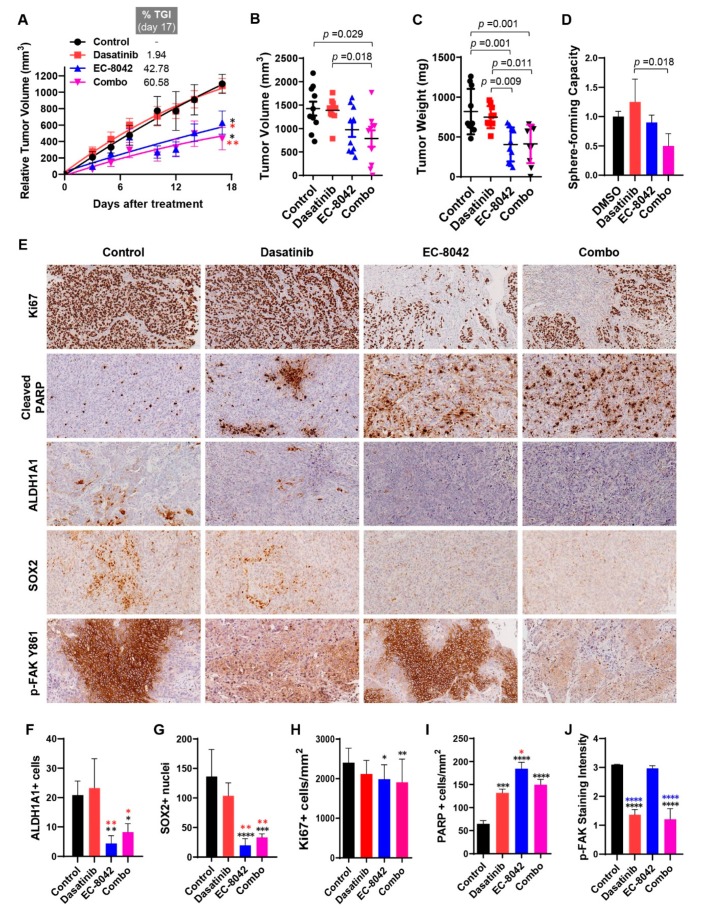
Effect of combination treatment with dasatinib and EC-8042 in FaDu xenografts. Mice with established FaDu xenografts were randomly assigned to four different treatment groups (*n* = 10 tumors per group) and treated with saline buffer intravenously (i.v.) and/or tartaric acid (orally) (control), dasatinib (orally) at a dose of 10 mg/Kg every day (16 doses), EC-8042 (i.v.) at a dose of 50 mg/Kg every 7 days (4 doses), or the dasatinib plus EC-8042 combination (combo). Animals were sacrificed 4 h after the last treatment with dasatinib and 24 h after treatment with EC-8042. (**A**) Curves representing the mean tumor volume of FaDu xenografts during the treatments. Drug efficacy expressed as the percentage of tumor growth inhibition (%TGI) at the end of the experiment is indicated. Mean ± SD (*n* = 10 per treatment group), RM-one-way ANOVA, Tukey’s test, * *p* < 0.05, ** *p* < 0.01 vs. control (black) and dasatinib-treated mice (red). (**B**,**C**) Distribution of tumor volumes (**B**) and tumor weights (**C**) at the end of the experiment. Mean ± SD (*n* = 10 per group), one-way ANOVA, Tukey’s test, *p* < 0.05 between the indicated groups. (**D**) For the evaluation of CSC subpopulations after drug treatments, xenograft tumors were harvested and dissociated into single cells to assess the tumorsphere-forming ability after in vivo drug treatment. Mean ± SD (*n* = 3 per group), one-way ANOVA, Tukey’s test. (**E**) Representative images and (**F**–**J**) graphs of the immunohistochemical analysis of Ki67, cleaved PARP, ALDH1, SOX2, and phospho-FAK (Y861) in paraffin-embedded tumors from FaDu xenografts after in vivo treatment with the indicated drugs. Scale bars = 100 μm. Mean ± SD (*n* = 5 per group), one-way ANOVA, Tukey’s test, * *p* < 0.05, ** *p* < 0.01, *** *p* < 0.001, **** *p* < 0.0001 vs. control (black), dasatinib-(red) or EC-8042-treated (blue) mice.

Importantly, EC-8042 and the combined treatment demonstrated a profound antitumor activity in vivo on FaDu xenografts compared to vehicle- and dasatinib alone-treated groups (Figure 5). Thus, treatment with EC-8042 or EC-8042 plus dasatinib led to a significant reduction of tumor volume growth, presenting TGI percentages of 43% and 61%, respectively, and also a significant increase in survival (Figure 5A,B and Supplementary Figure S8A,B). Likewise, tumor weights in vehicle and dasatinib series doubled those of EC-8042 and combination series (Figure 5C) and even the combination regime was able to produce tumor regression in one case (Supplementary Figure S8A). Notably, none of the treatments caused loss of weight (Supplementary Figure S8C) or any other adverse effects in treated mice. In any case, a two-way ANOVA analysis of the relative tumor volumes at the experimental end point (Figure 5B) or the tumor weights (Figure 5C) show that the interaction between dasatinib and EC-8042 is considered not significant (Supplementary Figure S6B–D) and therefore, no synergistic effect was expected [40] in line with the CI values calculated in in vitro assays.

The ability of drugs to target CSC subpopulations was also examined after in vivo treatments. Thus, a cohort of xenograft tumors was harvested at the end of the treatments, disaggregated into single cells and assayed for tumorsphere formation. Whilst tumorsphere formation slightly increased in dasatinib-treated tumors, we found an inhibition of tumorsphere-forming ability in tumors treated with EC-8042 alone, which reached statistical significance in combination with dasatinib (Figure 5D). Furthermore, immunohistochemical analysis also confirmed a significant decrease in the expression of both CSC markers ALDH1A1 and SOX2 in EC-8042-treated and combo-treated tumors (Figure 5E–G). We also found that EC-8042 and combination treatment led to a significant decrease in the percentage of Ki67-positive cells, as well as a significant increase in apoptotic cell death (cleaved-PARP staining) (Figure 5E,H,I). In addition, FAK (Y861) phosphorylation levels significantly diminished in dasatinib- and combo-treated tumors, as expected (Figure 5E,J). Taken together, these results demonstrate that EC-8042 and combination treatment efficiently targeted CSC properties and showed robust in vivo anti-tumor effects mainly due to reduced cell proliferation and increased apoptotic cell death.

Overall, we show evidence that HNSCC cells treated with a combination of dasatinib and EC-8042 benefit from favorable anti-tumor properties of both drugs while preventing the adverse pro-stemness effects induced by dasatinib. Even though our findings indicate the presence of additive cytotoxic effect in vitro but not in vivo synergistic effect, the combined treatment keeps the ability of dasatinib to abrogate relevant pro-tumor signaling (i.e., SRC/FAK signaling) and abolish invasive potential, while adding the potent capacity of EC-8042 to target CSC subpopulations and inhibit tumor growth, without any noticeable adverse interactions of both agents.

## 4. Discussion

Dasatinib and saracatinib have proven powerful antitumor activity in preclinical settings but rather limited clinical efficacy in cancer patients [14,15,16,21,22]. In the context of HNSCC, both drugs have failed to demonstrate any significant activity as single agents in patients with recurrent and/or metastatic disease [24,25]. We herein provide interesting new data uncovering important deleterious activities of dasatinib and saracatinib sustaining stem cell-like properties in HNSCC cell lines, which could be a plausible underlying reason to explain their lack of clinical efficacy as monotherapeutic agents in HNSCC patients.

Complete eradication of tumors requires therapies able to effectively eliminate the CSC subpopulations responsible for treatment resistance, relapse, and metastasis [41,42,43]. Indeed, we found that treatment of HNSCC cells with EC-8042, a mithramycin analog with reported anti-stemness activity in sarcomas [27], resulted in a potent elimination of both adherent cell cultures (bulk tumor population) and CSC-enriched tumorsphere cultures and demonstrated a robust antitumor activity in vivo in HNSCC xenograft models. EC-8042 was found to induce its anti-oncogenic effects through the inhibition of factors like SP1 [27]. In this regard, it has been demonstrated that SP1 is frequently upregulated in HNSCC and that a combined inhibition of SP1, using mithramycin, and TGFβ pathways induced cell death and prevented HNSCC recurrence [44].

In marked contrast to EC-8042, dasatinib completely abolished cell invasion but induced only a modest anti-proliferative effect in adherent HNSCC cultures. More importantly, dasatinib failed to eliminate CSC-enriched tumorspheres or to prove any significant antitumor activity in FaDu xenografts. In this line, it has been reported that dasatinib worsened the anti-tumor effects in combination with cetuximab or cetuximab and radiation therapy in FaDu-derived tumors [45]. In addition, saracatinib did not demonstrate a significant effect on HNSCC tumor growth in a mouse orthotopic model of tongue squamous cell carcinoma but impaired perineural invasion and cervical lymph node metastasis [46]. Similarly, results from a randomized trial aimed to test the treatment with erlotinib, dasatinib, or combination treatment in patients with operable HNSCC showed that erlotinib but not dasatinib significantly reduced tumor size [47].

The study of the effect of dasatinib and EC-8042 combination in HNSCC models shows non-synergistic but complementary effects, where both drugs exert complementary anti-tumor properties without antagonizing each other. Remarkably, combined treatment of dasatinib with EC-8042 efficiently counteracted the deleterious effects of dasatinib on CSC properties, thus showing potent anti-proliferative, anti-stemness, and anti-invasive effects in HNSCC cells and significant antitumor activity in xenografts. Clonal sphere-forming ability and also expression of well-known CSC markers were consistently and dramatically reduced both in vitro and in vivo by treatment with dasatinib and EC-8042, compared to dasatinib alone. Our results also evidenced additional beneficial effects of combination treatment, such as combined targeting of several factors and signaling pathways which are effectively inhibited by EC8042 (i.e., ALDH1, SOX2, NANOG, NOTCH1) and/or dasatinib (i.e., SRC/FAK/ERK and PI3K/AKT pathways), as well as complementary anti-proliferative, anti-stemness, and anti-invasive effects provided by each compound. It is therefore plausible that combined targeting of these signaling pathways may result in reducing more effectively the CSC subpopulations within the tumors.

The in vivo data clearly indicate that the reduction of tumor volume and weight observed appears to be caused mainly by EC-8042. According to our in vitro data, this is likely due to the potent anti-proliferative and cytotoxic effect of EC-8042 on both bulk tumor cells as well as CSC subpopulations. Contrasting this, dasatinib but not EC-8042 demonstrated robust anti-migratory and anti-invasive properties, and this beneficial effect was also observed by combined treatment in 3D spheroid invasion assays, probably mediated by inhibition of SRC/FAK signaling pathway and/or EMT. Notably, p-FAK Y861 was a major target of dasatinib but not EC-8042. Accordingly, p-FAK Y861 levels were found to robustly diminish by dasatinib or combo treatment both in vitro and in vivo. In marked contrast, p-FAK Y861 levels were not significantly affected by EC-8042 in vivo and only partially reduced in vitro, whereas p-FAK Y397 as well as total FAK levels were mainly targeted and robustly decreased by EC-8042. It seems quite reasonable to infer that this could exert a major influence on the ability of tumors to invade and metastasize. Altogether, these data suggest that patients treated with the combined treatment may benefit from the cytotoxic and anti-stemness effect of EC-8042 together with the prevention of cancer cell migration and invasion due to dasatinib action. These properties could be of particular importance for patients with advanced stages of disease who are at a higher risk of metastatic dissemination.

In contrast to these findings, it has been reported that dasatinib was able to inhibit Sox2 expression and tumorsphere formation in NSCLC cells [48,49]. These differences could be related to differences in cell and/or tissue context, since we consistently observed that dasatinib reduced the phosphorylation levels of SRC and also AKT whilst increasing endogenous Sox2 levels in HNSCC cells and also Sox2/Oct4 reporter activity. This highlights the need to establish accurate markers of drug response and adequate patient stratification to better select the patients who may benefit from the treatment with dasatinib.

## 5. Conclusions

This study unveils undesirable stem cell-promoting functions by dasatinib and saracatinib that could explain the lack of clinical efficacy of both drugs in HNSCC patients. Supporting this hypothesis, we show that HNSCC cells treated with a combination of dasatinib and EC-8042 benefit from complementary anti-invasive, anti-proliferative and anti-CSC functions. This combination counteracts the adverse pro-stemness effects induced by dasatinib and is therefore suggested as a novel therapeutic strategy for clinical testing in HNSCC patients. According to these data, novel combinational strategies with EC-8042 could contribute to improving treatment efficacy and long-term clinical outcomes in HNSCC patients.

## Supplementary Materials

The following are available online at https://zenodo.org/record/3358610#.XUQDjRSYOpo. Supplementary Methods. Supplementary Table S1: Primers used for real-time RT-PCR. Figure S1: Western blot analyses and cell viability of FaDu and UT-SCC38 cells treated with either DMSO (vehicle), 0.1 µM dasatinib or 1 µM saracatinib. Figure S2: Analysis of the expression of the CSC markers ALDH1A1 and SOX2 in CSC-enriched tumorspheres and adherent cultures of FaDu and UT-SCC38 cells by Western-blot and RT-qPCR. Figure S3: Quantification of the infrared fluorescent signals from the Western blot analyses shown in Figure 2C and Figure 4F. Figure S4: Effect of dasatinib and saracatinib on the expression of EMT markers in HNSCC-derived cell lines. Figure S5: Quantification of the infrared fluorescent signals from the Western blot analyses shown in Figure 4A. Figure S6: Analysis of synergism between Dasatinib and EC-8042. Figure S7: Effect of dasatinib, EC-8042 and combination treatment on the targeting of CSC subpopulations in HNSCC cell lines monitored by flow cytometry using the SORE6 reporter system. Figure S8: Pictures of tumors extracted from FaDu xenografts, in vivo survival analysis and mean body weight of mice during the in vivo treatments. Video S1: 3D spheroid invasion assay in FaDu cells treated with DMSO (vehicle). Video S2: 3D spheroid invasion assay in FaDu cells treated with 0.1 µM dasatinib. Video S3: 3D spheroid invasion assay in FaDu cells treated with 0.1 µM EC-8042. Video S4: 3D spheroid invasion assay in FaDu cells treated with 0.1 µM dasatinib plus 0.1 µM EC-8042.
